# Investigating Flubendazole as an Anthelmintic Treatment for Guinea Worm (*Dracunculus medinensis*): Clinical Trials in Laboratory-Reared Ferrets and Domestic Dogs in Chad

**DOI:** 10.4269/ajtmh.21-1222

**Published:** 2022-02-28

**Authors:** Christopher A. Cleveland, Kayla B. Garrett, Erin K. Box, Alec T. Thompson, Ellen K. Haynes, Deborah L. Elder, Robert L. Richards, Ania A. Majewska, Sarah Anne J. Guagliardo, Ryan E. Wiegand, John A. Bryan II, Fernando Torres-Velez, Karmen Unterwegner, Mario Romero, Hubert Zirimwabagabo, Metinou Sidouin, Philip Tchindebet Oaukou, Mbang Mahammat Ada, Bongo Nare Richard Ngandolo, Charles D. Mackenzie, Timothy G. Geary, Adam J. Weiss, Michael J. Yabsley

**Affiliations:** ^1^ University of Georgia, Athens, Georgia;; ^2^ Louisiana State University, Baton Rouge, Louisiana;; ^3^ Emory University, Atlanta, Georgia;; ^4^ The Centers for Disease Control and Prevention, Atlanta, Georgia;; ^5^ Zachery Consulting LLC, Ila, Georgia;; ^6^ The Carter Center, Atlanta, Georgia;; ^7^ The Carter Center, N’Djamena, Chad;; ^8^ Programme National d’Eradication du Ver de Guinée, Ministry of Health, N’Djamena, Chad;; ^9^ Institut de Recherche en Elevage pour le Développement, Afrique One Aspire, N’Djamena, Chad;; ^10^ NTD Support Center, Task Force for Global Health, Atlanta, Georgia;; ^11^ McGill University, Montreal, Canada;; ^12^ Queen’s University, Belfast, Northern Ireland

## Abstract

*Dracunculus medinensis* (Guinea worm [GW]), a zoonotic nematode targeted for eradication, has been managed using interventions aimed at humans; however, increases in domestic dog GW infections highlight the need for novel approaches. We conducted two clinical trials evaluating the efficacy of subcutaneously injected flubendazole (FBZ) as a treatment of GW infection. The first trial was conducted administering FBZ to experimentally infected ferrets; the second trial involved administering FBZ or a placebo to domestic dogs in the Republic of Tchad (Chad). We found contrasting results between the two trials. When adult gravid female GW were recovered from ferrets treated with FBZ, larvae presented in poor condition, with low to no motility, and an inability to infect copepods. Histopathology results indicated a disruption to morulae development within uteri of worms from treated animals. Results from the trial in Chadian dogs failed to indicate significant treatment of or prevention against GW infection. However, the difference in treatment intervals (1 month for ferrets and 6 months for dogs) or the timing of treatment (ferrets were treated later in the GW life-cycle than dogs) could explain different responses to the subcutaneous FBZ injections. Both trials provided valuable data guiding the use of FBZ in future trials (such as decreasing treatment intervals or increasing the dose of FBZ in dogs to increase exposure), and highlighted important lessons learned during the implementation of a field-based, double-blinded randomized control trial in Chadian dogs.

## INTRODUCTION

*Dracunculus medinensis* (Guinea worm [GW]) is targeted for eradication and has effectively been managed in humans via focal interventions such as provisioning clean water, water filtration, and the use of temephos (Abate) (to decrease intermediate host populations). The recent increase in dog infections with GW, particularly in Chad, provides new challenges to the eradication campaign, necessitating novel interventions and management approaches.^1^ Currently, interventions targeting dogs involve a reward system for dog owners who report infections, monitoring of symptoms consistent with preemergent and emergent GW infections by local health officials, and prolonged tethering by owners to prevent dog contact with bodies of water. However, village-level adherence to prolonged tethering may decrease over time, leading to inconsistent prevention of GW transmission through this method and requiring additional intervention approaches. To date, chemotherapeutic options have been unsuccessful in preventing or treating GW infections.^2^^–^^5^ Two previous, unpublished field studies in dogs investigating applications of oral ivermectin (Heartgard) and topical moxidectin (Advocate) provided no evidence of effective transmission interruption, warranting investigation of an alternative compound (The Carter Center Guinea Worm Eradication Program, unpublished).

Flubendazole (FBZ) is an anthelmintic that has shown success as a treatment of human neglected tropical diseases caused by parasitic worms, including onchocerciasis (*Onchocerca volvulus*) and lymphatic filariasis (*Brugia malayi, Brugia timori, Wucheria bancrofti*).^6^^,^^7^ FBZ has also been used in the treatment of gastrointestinal nematodes in humans, suggesting that this treatment may be useful for other nematode infections such as GW infection.^8^^,^^9^ Animal models have been used to evaluate oral formulations of FBZ as a treatment of filariasis, and to compare the efficacy of different administration routes (subcutaneous [SQ] injection versus oral formulation) of FBZ.^10^^,^^11^ Multiple studies have investigated the use of parenteral FBZ as a macrofilaricidal treatment in humans and animals.^12^^–^^17^ These trials were successful in decreasing the overall burden of parasites and supported the potential of FBZ as a macrofilaricide when administered in multiple doses or single high doses. Given its slow release from a depot site when given as a SQ injection, FBZ may be effective in preventing and treating GW infections in domestic dogs.^11^ Specifically, FBZ could prevent GW emergence by targeting various points in the life cycle, including infectious third-stage larvae (L3) within a copepod ingested by a definitive host, L3s within the musculature of a paratenic host ingested by a definitive host or developing L4 and young adult parasites.

The objectives of this work were to 1) investigate the effect of subcutaneously administered FBZ on GW infections in a model animal system (laboratory reared ferrets, *Mustela putorius furo*) and 2) evaluate the effect of SQ FBZ on GW infections among peridomestic dogs (*Canis lupus familiaris*) via a double-blinded randomized clinical trial in Chad.

## MATERIALS AND METHODS

### Drug formulation.

FBZ was acquired as a powder from TCI America (Portland, OR), and from Janssen Pharmaceutical (Beerse, Belgium). A sterile aqueous formulation of FBZ (250 mg/mL) was produced using purified United States Pharmacopeia (USP)-grade water, FBZ, 0.375% w/v hydroxyethylcellulose (Merck KGaA, Darmstadt, Germany), and 0.1% Tween 80 (Merck KGaA). The formulation was aseptically prepared under USP chapter 797 guidelines, and the current good manufacturing practices (cGMP) at the University of Georgia (UGA) College of Pharmacy (Athens, GA).

A working dilution (15 mg/mL) for injection was produced using an aqueous diluent of 0.375% w/v hydroxyethylcellulose and 0.1% v/v Tween 80 in sterile water. The diluent was sterilized using a standard autoclaving protocol for liquids. The sterile diluent was used to prepare the formulation via an Unguator electronic mortar (PCCA, Houston, TX) and certified laminar flow hood following USP chapter 797 and cGMP guidelines.

For the dog trial, the placebo was Intralipid 20% (Fresenius Kabi, Uppsala, Sweden), a sterile, nonpyrogenic fat emulsion consisting of 20% soybean oil, 1.2% egg yolk phospholipids, 2.25% glycerin, and sterile water. This product was selected because it most closely mimicked the look and consistency of the FBZ formulation, thus facilitating the blinded administration of these treatments in the field.

### Laboratory FBZ trial in experimentally exposed ferrets.

#### Infection and treatment of ferrets.

Fifteen laboratory-reared ferrets (Triple F Farms, Gillett, PA) were exposed to *D. medinensis*–infected copepods (100/ferret) per os in November 2018 using previously described methods.^18^ Computed tomography (CT) and ultrasound were conducted at the University of Georgia College of Veterinary Medicine (Athens, GA) by a board-certified veterinary radiologist to check for evidence of infection at 8 months postexposure. At 9 months postexposure when migrating adult worms are appreciable, six ferrets were identified as having SQ worms based on palpation: five animals were designated to receive FBZ (15 mg/kg SQ), and one was designated as a control. Two treatments were administered 30 days apart (August and September 2019), and each treatment involved a single SQ injection of FBZ on the proximolateral aspect of the left leg daily for 3 consecutive days. All ferrets were weighed before treatment administration to calculate the appropriate injection volume of the FBZ formulation described earlier.

#### Postexposure assessment and worm extraction.

Between 304 and 379 days postexposure (dpe), ferrets were euthanized under deep anesthesia (intramuscular ketamine [30 mg/kg] and xylazine [2 mg/kg]) using an intracardiac injection of sodium pentobarbital (1 mg/kg, Beuthanasia, Merck Animal Health, Madison, NJ). Necropsies were performed immediately after euthanasia. For each ferret, the total number of worms and location of each worm were recorded, then each worm was isolated from the ferret tissues, placed in a 50-mL conical vial filled with 30 mL dechlorinated water, and observed for the release of larvae. Within an hour of placement in water, larvae were removed from their respective vials and checked for motility and any morphological disruptions that could be attributed to FBZ treatment. If recovered first-stage larvae (L1) appeared viable and displayed sinusoidal motility, they were added to a petri dish containing 100 to 154 cyclopoid copepods collected from Chad. Copepod consumption of larvae and infection status of larvae within copepods were evaluated 24, 48, and 72 hours later. Larval maturation to the L3 stage was made 14 days after copepod exposure to assess the ability of larvae to mature within the cyclopoid copepod intermediate host. All observations were conducted using dissecting and compound microscopes.

#### Histopathology of worms from FBZ-treated ferrets.

To evaluate histological changes in *D. medinensis* worms in response to FBZ treatment, 2.0- to 2.5-cm segments were selected randomly from five female *D. medinensis* worms isolated from FBZ-treated ferrets, the control ferret, and an additional five worms from untreated animals from previous work.^19^ Sections were fixed and stored in 10% formol (3.7% formaldehyde), then embedded using HistoGel specimen processing gel (Thermo Scientific, Waltham, MA) allowing for the production of transverse sections through the worm segments.^20^ Sections were cut from three levels (∼100 μm apart) of each worm segment and stained with hematoxylin and eosin. Each section was first assessed for histological integrity before scoring for any damage or degeneration in any of the major anatomic components of worms, including uterine contents.

### Field trial with FBZ in domestic dogs, Chad.

#### Study design and site selection.

To ensure sufficient sample sizes for statistical analyses, an initial set of 600 dogs was randomized to receive SQ injections of FBZ (*N* = 300) or SQ placebo injection (*N* = 300). As in the ferrets, each dog treatment involved one injection per day for 3 consecutive days.

**Figure 1.**
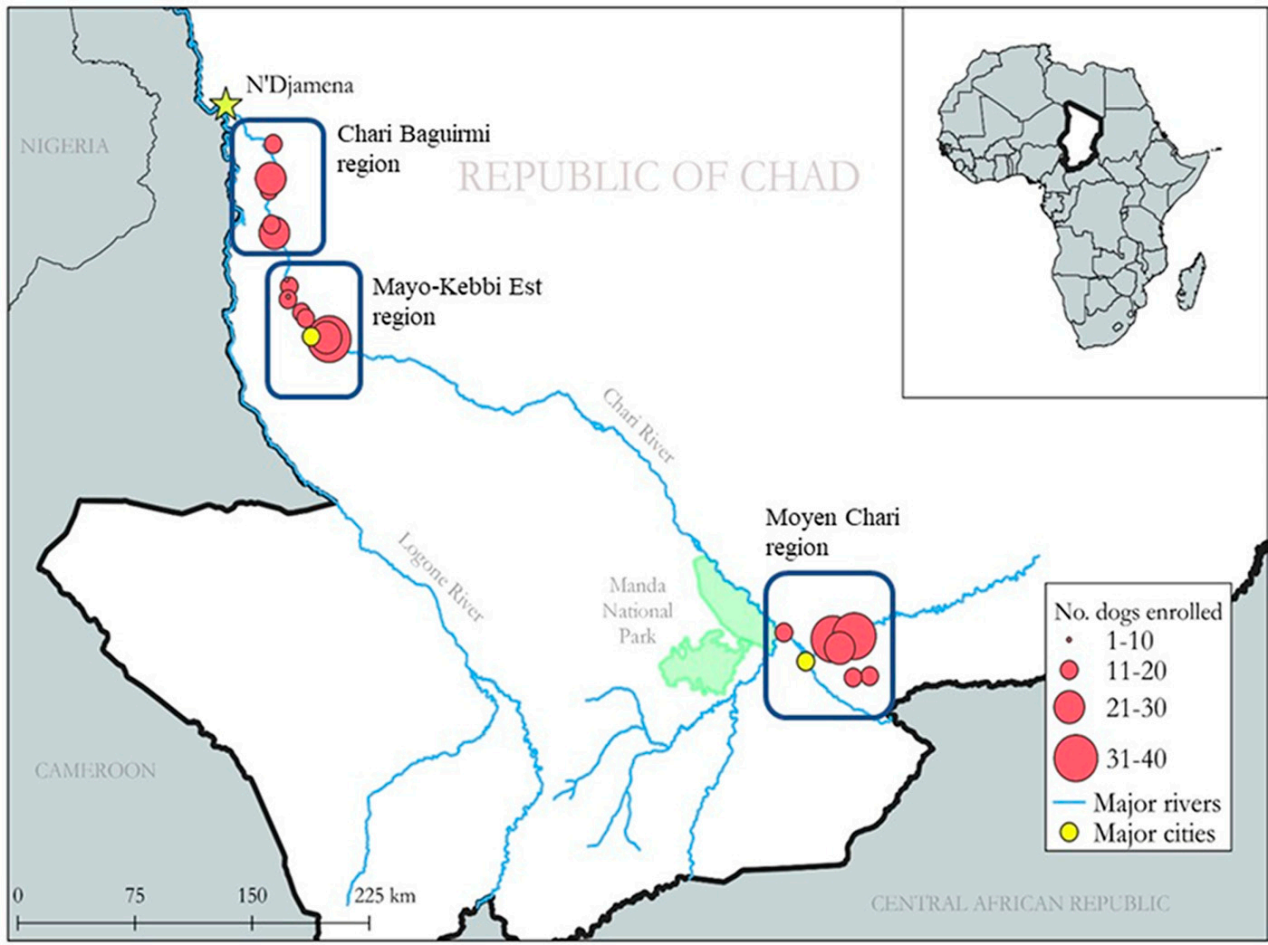
Study regions, sites and approximate numbers of dogs enrolled in each village for the FBZ randomized clinical trial, Chad. This figure appears in color at www.ajtmh.org.

Dogs were enrolled from 23 villages in Chad (Figure 1, Table 1). Study sites were located in three known foci of canine GW transmission in the northwest Chari region (Chari Baguirmi and Mayo-Kebbi Est Regions) and the southeast Chari region (Moyen Chari Region) (Figure 1, Table 1). Among the 548 villages reporting GW in dogs from 2015 to 2018, villages with higher GW prevalence in dogs were prioritized to maximize the number of dogs enrolled per village, thereby reducing the need to traverse long distances between study sites. Specifically, the 2018 period prevalence of canine GW was estimated at the village level using infection counts from routine surveillance and dog population estimates from an annual dog census conducted by The Carter Center personnel in January 2019. Of 548 possible villages in the regions of interest, we excluded villages with no dog infections detected in 2018 (*N* = 263), villages with ongoing studies (*N* = 7), and villages that were either difficult to access or had no dogs (*N* = 248), leaving 30 possible sites. Once in Chad, 23 villages were selected based on reliable accessibility across seasons for the entirety of the study.

**Table 1:** Demographics of study sites chosen for inclusion in the flubendazole randomized clinical trial in domestic dogs, Chad Africa

Region	Village	Human population (2018)	Estimated dog population (2018)	Estimated percent prevalence of canine Guinea worm (2018)*
Chari-Baguirmi	Aligarga Sara	208	8	100
	Godogo	313	26	30.77
	Darda	750	58	24.14
	Raf Center	942	38	21.05
	Abachere	248	25	20
	Mecontie	259	60	18.33
	Bougoumene	1,243	106	9.43
	5 Kilo	437	36	8.33
Mayo-Kébbi Est	Abory Mbarma	604	23	21.74
	Boudanassa Lambe	505	103	11.65
	Mogrom Center	1,335	26	11.54
	Mogrom Biao	762	29	10.34
	Boudanassa Bao	188	31	9.68
	Magrao	810	93	7.53
	Sawata	565	31	6.45
	Kakele Mberi	720	150	6
Moyen-Chari	Kaimamba	156	32	25
	Dankolo	468	51	13.73
	Doumabe	265	26	11.54
	Bodobo 2	510	40	10
	Dangala Kanya	301	63	9.52
	Marakouya 2	428	85	9.41
	Marabe 2	420	300	7

* Percentage of dogs infected during 2018.

#### Dog qualification.

Dogs were selected for study inclusion based on assessments of disposition, age, physical condition, and owner consent. Assessments were conducted on site by project veterinarians upon arrival in each study village. Passing assessments were required to secure qualification. Owner consent was required for each dog enrolled in the study. Owners were provided an explanation of the study, highlighting aspects of participation in both written and oral forms. Once informed and their questions were answered, owners who agreed to participate signed the study consent form. Further details on dog qualification assessments are provided in the supplemental material.

#### Sample collection and FBZ administration.

Enrolled dogs were visited once per day for 3 consecutive days. Day 1 procedures included initial assessment, random assignment to placebo or treatment group, microchip placement, palpation for subcutaneous GW, blood sampling, rabies vaccination, and administration of FBZ or placebo (dose one of three). Days 2 and 3 procedures included second and third doses (FBZ or placebo administration), opportunistic fecal sample collection, and palpation for SQ GW. This 3-day round of treatment was repeated at two additional timepoints, each 6 months apart. Owners and treatment teams were blinded to the treatment that dogs received, a status maintained throughout the study.

For unique identification, dogs were injected with an SQ microchip (Avid2028 9-digit chips, 125 kHz, Avid Identification Systems, Norco, CA). A prefabricated sterile AVID delivery device (Avid Identification Systems) was used to inject the microchip subcutaneously between the shoulder blades on the dorsum of each dog. Injection sites were aseptically prepared using 70% to 90% EtOH. After placement, each microchip was verified by an associated AVID Mini Tracker I microchip reader (Avid 1002). Dog limbs were palpated and visually inspected by the project veterinary team to determine the presence of discernible subcutaneous GWs. Detected worms were counted, and their anatomic locations were recorded, photographed when feasible, and reported to Program National d’Eradication du Ver de Guinée (PNEVG) personnel. For blood collection, venipuncture sites were prepared with 70% to 90% EtOH, and a blood sample (∼0.7 mL) was collected from the cephalic vein. Blood collected in the field was placed in 3-mL EDTA vacutainers (Becton, Dickinson and Company, Franklin Lakes, NJ) and stored upright in a field cooler with a frozen ice pack. Upon returning to the centralized field laboratory, a 125-μL aliquot was transferred to a Whatman FTA card (Cytiva, Marlborough, MA) and 10-μL aliquots transferred to each tab (*N* = 6) of a paper disc (TropBio Pty Ltd, Queensland, Australia) for pathogen screening and serological assay development, respectively, to be conducted at a later date. After filter paper sampling, plasma was allowed to separate until visual differentiation of plasma from cells could be appreciated, then removed using a bulb pipette, aliquoted into 1.5-mL tubes and frozen at –20°C until shipment to the UGA. Doses of FBZ or placebo were administered subcutaneously on the proximolateral aspect of the left leg using a 20- to 22-gauge × 1-inch needle. Injection sites were aseptically prepared using 70% to 90% EtOH. Laboratory staff at UGA were not blinded to treatment condition (FBZ versus placebo).

#### Follow-up assessments of dogs and worms.

Village-level supervisors conducted follow-up assessments on dogs enrolled in the FBZ trial a minimum of twice per month. At each follow-up visit, the dog was identified using the microchip scanner and received a visual examination and manual palpation for subcutaneous worms, as well as any developing lesions/infections. If a dog was identified with lesions consistent with GW infection (e.g., presentation of a blister, swelling, subcutaneous worm that had not yet emerged, or wound), it was tethered and received daily monitoring per current PNEVG guidelines.

To evaluate the effects of FBZ on development of larvae within gravid female worms in Chadian dogs, worms removed from dogs were immediately placed in water to observe larval motility. Female worms were placed in Petri dishes containing water from a clean source free of copepods, such as well water or bottled water. Petri dishes with worms were allowed to sit for 10 min to allow larvae to emerge from the worm; if no larvae were observed, the worm was cut to release any larvae present. Using a handheld microscope (Carson MicroFlip 100× to 250×, Carson Optical, New York, NY), larval movement was quantified as follows: 0 = no movement, larvae dead; 1 = very little movement, 75% larvae not moving; 2 = moderate movement, slow, 50% larvae not moving; 3 = good movement, good speed (larvae wiggling), 25% larvae not moving; or 4 = excellent movement, fast speed, 10% larvae not moving. Absence of larvae was also noted. After the larval assessment was complete, the female worm was placed in ethanol for subsequent identification.

#### Statistical analyses.

Statistical analyses and visualization assessing the effect of FBZ treatment on GW larval motility, number of worms per dog, and prevalence of worms in dogs were conducted in R (version 3.6.3).^21^ Data for analysis included the dog demographic information, treatment received, GW detection, and larval motility data from the FBZ trial described earlier. We used generalized linear mixed models implemented in the glmmTMB package for statistical analyses.^22^ Models were fit with main effects of dog treatment group, dog age, dog sex, treatment round, and the interaction between round and treatment group. We also included nested random effects of dog identity and village identity to control for random variation in response variables between dogs and villages. Response variables were modeled using the distribution determined for best fitting based on inspection of diagnostic plots: larval motility (motile versus nonmotile; binomial), binary presence of GW detection in a dog (prevalence; binomial), and number of GW detections per dog (intensity; negative binomial). Analysts were not blinded to treatment condition.

## RESULTS

### FBZ in experimentally exposed ferrets.

At 8 months postexposure, CT scans and ultrasound detected no evidence of infection in ferrets. The first evidence of infections in exposed ferrets was at 9 months postexposure (August 2019) via visual inspection and palpation of subcutaneous worms located in the distal extremities. Of 15 exposed ferrets, six were confirmed to be infected with GW using these methods. Of note, three ferrets (ferrets 2, 3, and 4; description of cases provided in the supplementary materials) developed sterile lesions, measuring approximately 2 × 2 cm, at injection sites 7 to 9 days after the second treatment. This was attributed to the short duration between FBZ injections given at the same administration site. Affected ferrets were treated with a 10-day course of cephalexin (25 mg/kg by mouth twice daily) and lesions resolved within 1 month of appearance.

Infected ferrets treated with FBZ were euthanized between 10 and 12 months postexposure (ferrets 1–5). Three ferrets (ferrets 1–3) had GWs that appeared mature and gravid on necropsy (Table 2). When larvae were allowed to emerge in water and their behavior was observed, their overall motility (swimming movement) was impaired and sluggish (motility scale 0–1). In one case, some embryos were not fully developed, and those that were fully developed first-stage larvae lacked the swimming vigor associated with normal, healthy GW larvae. The remaining two ferrets (ferrets 4 and 5) were infected with worms that had no viable larvae to assess (Table 2). Copepods exposed to larvae recovered from FBZ-treated ferrets showed a < 1% infection rate (seven infected copepods of 399 exposed), compared with a 15% to 30% infection rate in copepods using healthy larvae from the control ferret (Table 2).

**Table 2:** Necropsy timing, adult worm recovery, and the ability of recovered larvae to infect copepods and develop to third-stage larvae (L3s) for GW experimental infection and FBZ treatment trial among laboratory reared ferrets

Ferret	Necropsy timing (dpe)	# Female worms recovered	Proportion of infected/exposed copepods	# L1 that matured to L3 in copepods
Control	304	4	31/100	29
1	324	2	6/100	1
2	331	1	1/145	0
3	350	1	0/154	0
4	371	1	* NA	*NA
5	379	2	*NA	*NA

* NA = no larvae were recovered from GW found in FBZ-treated ferrets.

#### Histopathology of FBZ worms from treated ferrets.

**Figure 2.**
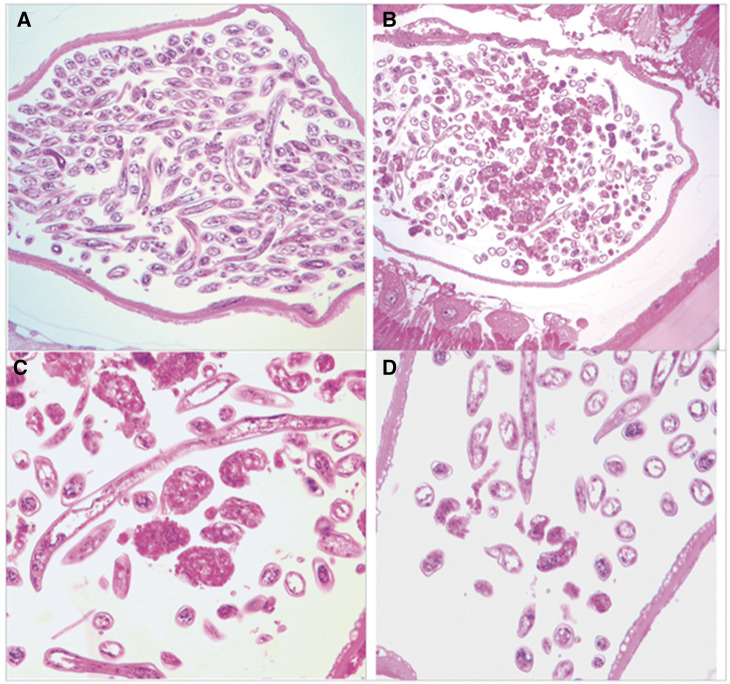
Images of the uterine components of *Dracunculus medinensis*. (**A**) Healthy uterus of a worm isolated from an untreated animal. (**B**) Damage to larval forms within uterus of worm. (**C**, **D**) Uterine contents from a worm taken from a FBZ-treated animal showing damage to morulae. This figure appears in color at www.ajtmh.org.

With the exception of the uterus, all other major anatomic structures of worms from ferrets receiving FBZ or no treatment/placebo were free of any marked changes or abnormalities. No marked anatomic changes were seen in the body wall of any of the worms in either major group. Uterine wall damage and degenerative changes were seen in the worms from the FBZ-treated animals; these changes consisted of loss of membrane integrity, an increase in uptake of stain, and a consequent loss of normal form in the cells lining the uterus. The number of developing forms present in the uterine lumen varied considerably, attributed to technical problems in worm extraction and preparation. Overall, there were considerably fewer developing larval forms present in the worms from animals treated with FBZ than were present in the parasites recovered from the untreated ferret (Figure 2A and B). Most of the developing forms in the FBZ group were damaged or degenerating to varying degrees, the most significant changes being the presence of degenerating morulae and poorly developed larval forms (Figure 2C and D). Degenerating larval forms were uncommon in the five worms isolated from untreated animals from previous work and the one control ferret, and damaged degeneration morulae were not observed in this group.

### FBZ efficacy in Chadian dogs.

A total of 435 dogs in 23 villages (Table 1) met study criteria and were enrolled in May 2019 (Table 3). The average age of dogs enrolled in the study was 2.5 years (range: 1–5 years), and average weight for all dogs across three rounds was 13.86 kilograms (kgs) (range: 8–23 kg). Dog loss to follow-up from round 1 to round 2 was 27%, from round 2 to round 3 was 25%, and overall loss to follow–up was 42% (Table 3). Randomization of dogs within each village (assigned to either FBZ treatment [*N* = 222] or placebo [*N* = 213]) ensured a nearly even split of dogs per village throughout the study.

**Table 3:** Demographic data of dogs enrolled in the three rounds of flubendazole trial, Chad

Round	Round 1: May 2019	Round 2: Nov 2019	Round 3: June 2020
N dogs	435	318	254
FBZ group	222	165	126
Placebo group	213	153	128
N males	262	194	154
N females	173	124	100
Average weight (kgs)	13.6	14	14
Loss to follow up from Round 1% (n)		27% (117)	42% (181)

**Figure 3.**
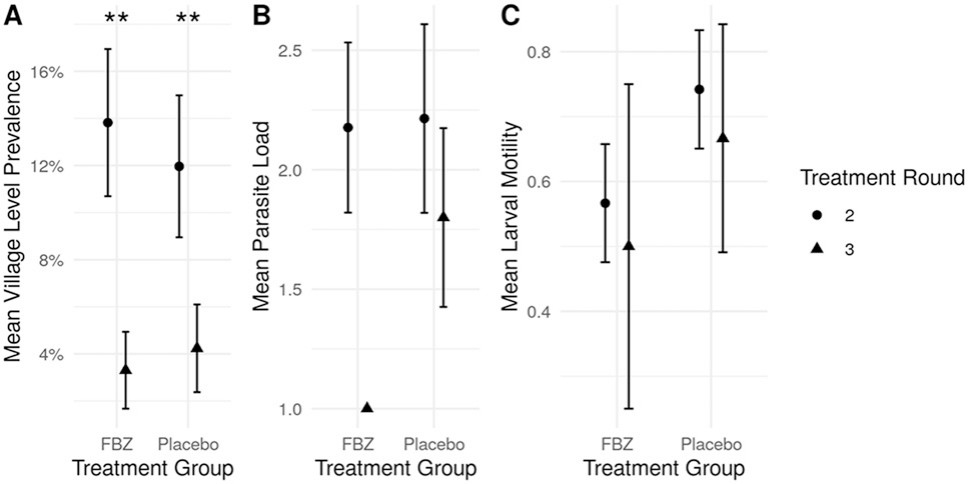
The effect of treatment group (FBZ vs. Placebo) and treatment round (2^nd^ vs. 3^rd^) on GW response to FBZ treatment. (**A**) Mean (±SE) prevalence of *D. medinensis*, (**B**) mean parasite load, and (**C**) mean proportion of larvae that show motility. Asterisks note significant relationships in generalized linear mixed models.

**Figure 4.**
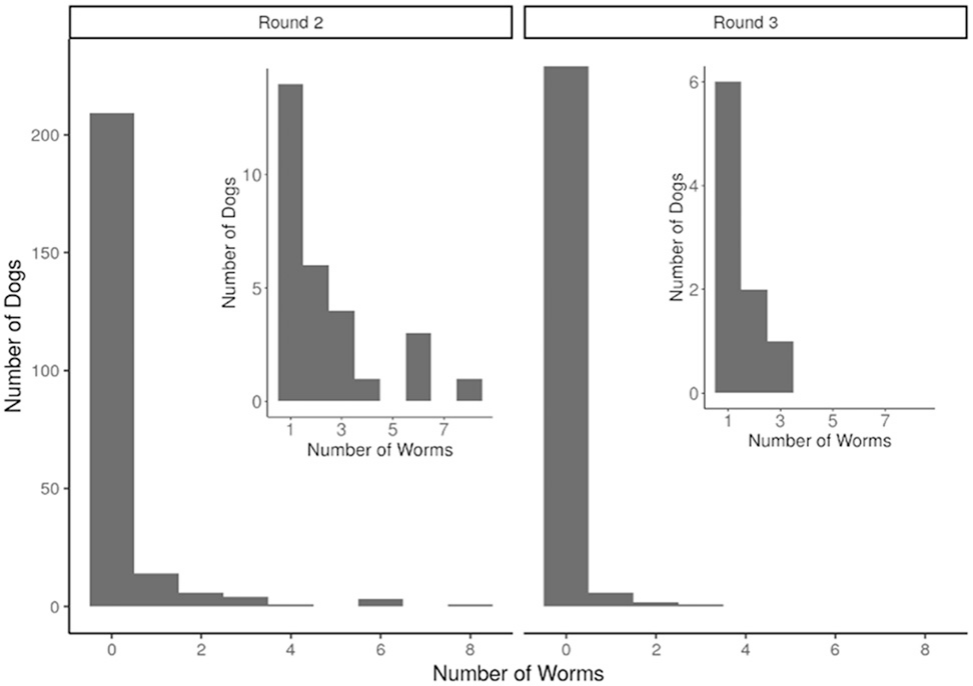
Number of *D. medinensis* nematodes detected per dog in rounds 2 and 3 of the FBZ trial. Insets represent the distribution of recovered worms (1–8) per dogs in rounds 2 and 3.

We found no significant difference in prevalence of *D. medinensis* infection between FBZ- and placebo-treated dogs, nor was the interaction between treatment group and treatment round significant (Figure 3A, Table 4). However, prevalence did significantly differ between treatment rounds, likely due to the seasonality of the parasite life cycle. Worm burdens did not significantly differ between treatment groups or between seasons, and the interaction between treatment group and season was not significant (Figure 3B, Table 4). The average number of worms per dog when infected between rounds was 1--3, with only a few dogs having a higher burden of infection (Figure 4). When *D. medinensis* worms were recovered from infected dogs and larval motility was assessed using a numeric scoring system, treatment group A (FBZ) had a slightly lower proportion of motile samples than treatment group B (placebo). This difference was not statistically significant, nor were any other modeled predictors, (Figure 3C, Table 4).

**Table 4:** Summary table for generalized linear mixed model of *D. medinensis* prevalence, burden, and larval motility. Statistics show the χ^2^ value, degrees of freedom, and *P* value (random effect variances are shown for village and individual dogs)

	Prevalence			Burden			Larval motility		
Variable	Χ^2^	df	*P* value	Χ^2^	df	*P* value	Χ^2^	df	*P* value
Treatment Group	0.077	1	0.782	0.4308	1	0.512	1.056	1	0.304
Round	5.841	1	0.016	1.797	1	0.180	0.007	1	0.935
Sex	2.366	1	0.124	1.113	1	0.292	1.956	1	0.162
Age	3.139	1	0.076	0.630	1	0.427	0.373	1	0.541
Treatment Group*Round	0.098	1	0.754	0.613	1	0.434	0.290	1	0.590
Random effects σ^2^									
Village	11.64			0			0.75		
Individual	0.3143			0			0		

## DISCUSSION

Our study examined the efficacy of FBZ as a preventive and/or treatment of GW infections using experimentally infected ferrets and naturally infected peridomestic Chadian dogs. Our aims were to examine the effects of FBZ on developing GW in a definitive host in a controlled laboratory setting and to assess the feasibility of a large-scale treatment program in definitive hosts where transmission and infection are endemic. The ferret and Chadian dog trials had different designs and together suggest that there are outstanding questions to be addressed regarding FBZ as a preventive or treatment intervention for GW infections. Specifically, the dose and treatment interval warrant further investigation, as discussed subsequently.

GW poses unique challenges in experimental infection trials, as highlighted in the ferret trial. The 10- to 14-month gestation period of GW, coupled with a lack of tests to confirm infection before patency, complicate achieving consistent infections in experimental animals. Moreover, limited data are available on the factors that lead to infection in the definitive host, such as inoculum-dependent responses for GW, which illustrates the relationship between the number of L3s placed in a definitive host and the number of adult nematodes recovered. For example, 15 ferrets were exposed to 100 infected copepods per os, yet only six ferrets developed patent infection with variable numbers of adult females recovered (range = 1 to 4; average = 1.8; Table 2). In future trials, it will be important to consider the unpredictable results of this experimental infection protocol.

The results of the ferret trial suggest that FBZ impacts the infectivity of GW larvae. The control ferret developed a patent infection with four gravid female GWs and larvae recovered from this ferret swam vigorously, with a 30% infection rate of exposed copepods and the majority of larvae molting to L3. In comparison, the five GW-infected, FBZ-treated ferrets had one or two recovered gravid females and the few larvae recovered from these worms displayed sluggish, halted movement and low rate of copepod infection and maturation to L3. Vigorous sinusoidal movement of larvae is integral to attracting copepods for subsequent ingestion for the obligatory larval molts from L1 to L3 before infection of the definitive host.

Worms recovered from the last two euthanized ferrets (ferrets 4 and 5 at 371 and 379 dpe, respectively) were in a state of degradation and necrosis and yielded no viable larvae, which might have been the result of prolonged exposure to FBZ. FBZ is a lipophilic compound, and at late-stage gestation just before patency, adult female GWs migrate through superficial fascial layers proximal to hypodermal adipose.^23^ Potential accumulation of FBZ in hypodermal adipose tissue could result in a more pronounced effect of FBZ on late-stage GWs and explain the observed mortality of recovered worms. Further work investigating the effect of FBZ on larval motility is warranted and was attempted during Chadian dog trial follow-up periods, as discussed subsequently.

The distinctive degenerative changes in uterine tissues and developing forms of the FBZ-exposed worms recovered from ferrets are consistent with the degenerative changes seen in other filarial nematodes exposed to FBZ.^15^^,^^20^ The commonly seen dissolution and degeneration of the rapidly developing new morula stage is likely due to the tubule disassociating property of FBZ. The high degree of damage to the morula and other uterine stages induced by FBZ is thought to cause blockage of the uterus with the consequent death of the adult worm. It is possible that higher FBZ doses than those used in the present study would lead to more extensive uterine and adult worm damage.

The initial experimental design for the Chadian dog trial was modified after the start of fieldwork, based on the finding that dog population counts in villages differed significantly from the PNEVG census report used for planning. The original experimental design targeted villages along the Chari River with historical GW infection prevalence of at least 15% in dogs and high dog populations, and the target sample size was 600 dogs across eight villages, split evenly between FBZ and placebo groups. This approach allowed for the strongest statistical analyses and accurate project assessments while concurrently minimizing the impacts of variables beyond the control of the study, including human behavior, feeding of dogs, and variations in dog management and behavior.

When the target dog sample size was not met in the preselected eight villages, the addition of more villages was required to achieve the highest possible enrollment of dogs. Ultimately, 23 villages (Table 1) were enrolled in the trial, resulting in 435 dogs that met a priori inclusion criteria. These inclusion criteria (assessments of affect, age, physical condition, and owner consent) were chosen to ensure owner consent, avoid enrolling older or unhealthy dogs that would be more likely to die during the study, and exclude dogs whose temperaments could endanger owners and study personnel during handling for treatment and sample collection. Treatment of dogs required tethering for 3 days, a requirement upheld with continuity during the study. One dog died during the 3 days of the first treatment from complications of tethering. Upon each visit to tethered dogs, project teams were encouraged to highlight the importance of provisioning food and water to the dogs and ensuring that dogs were tethered in a location with access to shade. Dogs treated with flubendazole did not exhibit any adverse effects to treatment: no blisters or sterile abscesses were noted on any dogs enrolled in this trial.

Data were analyzed between rounds, and for the entire duration of the trial to assess the efficacy of FBZ as a preventive and/or treatment of GW infection in domestic dogs. No significant effects of treatment were detected on prevalence or mean worm intensity. Although treatment with FBZ appeared to reduce larval motility compared with larvae recovered from the placebo group, this effect was not statistically significant and cannot be attributed to treatment. Additionally, the total number of dog infections reported from villages across the duration of the study did not decrease. Results indicate no other significant effects when evaluating prevalence, emergence, or infection intensity of GW as a result of treatment with FBZ in the tested regimen.

There were several differences in the study design between the ferret and dog trials that may explain the different outcomes. Detection of the infections at a later stage of development in the ferrets resulted in FBZ treatments being administered in a different regimen (two treatment rounds 1 month apart) than in the randomized clinical trial in dogs in Chad (three treatment rounds 6 months apart), although all animals received the same 15 mg/kg dose of FBZ. This was the best option to investigate whether FBZ has an effect on existing GW infections, but likely influenced systemic drug levels and impacts on *D. medininsis* worms. Given the anticipated long duration of plasma levels of FBZ due to slow leaching from the depot site, systemic drug levels likely had a higher peak in the ferrets compared with dogs.^13^ It is possible that this presumptive increased exposure of GWs to FBZ is responsible for the variable presentation of worms recovered from ferrets, as highlighted by the marked degradation of the female worms recovered from the two ferrets with the longest FBZ exposure (last to be euthanized).

Further investigation in a controlled experimental setting is needed to evaluate the differences of treatment with FBZ on early- versus late-stage infections with GW. The treatment interval is perhaps the most pronounced difference between the two trials, and begs further evaluation. The treatment regimen of injections every 6 months was based on evidence of FBZ slowly disseminating from a subcutaneous depot (Geary and Mackenzie, personal communication). Although a monthly treatment regimen may be challenging for a field project, it is feasible in a controlled laboratory setting where samples could be collected monthly to investigate the pharmacokinetics and pharmacodynamics of FBZ treatment. For example, FBZ may be more rapidly metabolized in dogs than in ferrets, producing a shorter-than-expected exposure duration of GW. It is also important to note that the dose chosen (15 mg/kg) was conservative, was chosen to minimize any negative effects from the SQ administration route to Chadian dogs, and that much higher doses have been administered parenterally to many mammalian species including dogs.^12^^,^^24^ Thus, injections with higher doses in dogs may generate the same level of efficacy observed in the ferrets, with significant impacts on GW transmission; however further work is needed to ensure safe administration to dogs and eliminate the possibility of abscess formation.

Large field trials have considerable logistical challenges, and this study provided important lessons learned that can be applied to future efforts. Acquisition and compounding of FBZ was straightforward; however, many details regarding stability once compounded remain unknown. To create a subcutaneous depot, FBZ is suspended in hydroxyethylcellulose, a compound that binds FBZ, allowing for prolonged systemic absorption after SQ injection. In the hot Chadian climate, the high viscosity of this formulation required large-gauge needles to fill syringes and made injection slow and difficult. This was addressed by keeping FBZ and placebo vials chilled in a cooler with ice packs without freezing, but this complicated field administration. During previous studies, unique identification of dogs was a challenge.^25^ In this study, the use of SQ microchips facilitated consistent data collection from and treatment administration to dogs who were available for inclusion in the entirety of this study. The treatment teams successfully administered Avid microchips to dogs, providing a unique identification system that ensured accurate follow-up. Training was provided to village-level supervisors on the use of the microchip scanners, a task instrumental for facilitating follow-up of dogs enrolled in the trial. Microchips, when applied correctly, are a low-cost, long-term, effective mechanism to identify dogs. It is highly recommended that this option be considered for future studies to avoid issues frequently encountered in the field, such as multiple names for a single dog, different spelling of dog names, and multiple owners per dog.

In conclusion, the results of this work, specifically the efficacy in the ferret model, indicate that FBZ may be a worthwhile anthelmintic treatment of infection with GW. However, additional work is necessary to identify key details, specifically the most effective dosage and frequency of administration, as well as the pharmacokinetic profile in dogs. Notably, this work has also shown that a large-scale treatment program targeting GW-infected dogs is possible in Chad and that village-level support and adherence to research trials involving dogs is high. The FBZ trial was yet another important step taken toward eradication of GW, and we anticipate that the lessons learned from the field and laboratory trials will further forthcoming work aimed at the eradication of Guinea worm.

## Supplemental Material


Supplemental materials
