# Supplementary material for: Investigating Flubendazole as an Anthelmintic Treatment for Guinea Worm (Dracunculus medinensis): Clinical Trials in Laboratory-Reared Ferrets and Domestic Dogs in Chad
Source: Am J Trop Med Hyg. 2022 Feb 28;106(5):1456–65. doi: 10.4269/ajtmh.21-1222 (PMC9128682; doi:10.4269/ajtmh.21-1222)
Supplement: Supplementary file 1 [file tpmd211222.SD1.pdf]

Supplemental Data:

Dog qualification assessment details:

The initial assessment focused on three aspects: *material*, *physical*, and *emotional*, and took place upon the first approach of a candidate dog. *Material* aspects were assessed via *yes* or *no* responses to questions, including whether the dog was present and attached/tethered; whether its chain and lock were in good working order; whether it had access to shade, shelter, and food and water; and whether the dog was tethered in such a manner as to leave it susceptible/vulnerable to harm from its own movements or other animals. *Physical* aspects were assessed via veterinary evaluation, and included determining whether a candidate dog was bright, alert, and responsive; had an acceptable nutritional plane; no signs of illness; and if female, whether lactating/nursing. *Emotional* parameters were assessed by veterinary evaluation and included the approachability (fractiousness) of candidate dogs in the context of handler safety.

The physical assessment included an age determination; established via the assimilation of an owner-reported age for the candidate dog and a direct examination of the dog's dentition. Candidate dogs with deciduous teeth were assumed to be less than one year of age and were thus disqualified. Dogs with advanced levels of tartar and/or wear were assumed to be toward the upper qualifying age limit for the study (five years) and were subsequently qualified or disqualified following supplemental assessments of conformation, nutritional status, and overall health. Dogs received a physical examination to assess overall physical conditions that would potentially disqualify a dog. Pregnant females were also excluded.

<u>Individual descriptions of Ferret exposures and treatment with FBZ</u>

Control ferret (E5037):

The control ferret was exposed to GW infected copepods in November 2018 and was euthanized in September 2019 (304 dpe). The ferret was infected with 4 mature, gravid female GW. Larvae were recovered, and exhibited rapid swimming behavior characteristic of normal, healthy GW larvae. When these larvae were exposed to copepods, we recorded a 15-30% infection rate in copepods; the variation in infection rate has to do with copepod mortality over time. Initial infection rates 72 hours after copepod exposure were 30% with a single first-stage larva present in copepods. Over the course of copepod maintenance, and allowing larvae to mature to L3s, copepod numbers decrease because of mortality. Four weeks post exposure to larvae, we recorded a 15% infection rate in remaining copepods and used these copepods to expose two new laboratory ferrets to GW.

Ferret 1 (E5031):

Ferret 1 received FBZ treatment, then was euthanized and necropsied on September 26, 2019 (324 dpe). We recovered two female GWs from the right front leg. Larvae appeared sluggish when observed under dissecting and compound microscopes. We exposed copepods to these larvae and found six copepods (out of 100) infected with a single GW larva. Of the six larvae, only one developed to the L3 stage as identified by the morphological characteristic of a blunt, trifid tail. The remaining larvae did not molt, retaining characteristics of L1s, and had died within the copepods.

Ferret 2 (E5039):

Ferret 2 received FBZ treatment, then was euthanized and necropsied on October 4, 2019 (331 days post exposure). Of note, this individual ferret developed sterile abscesses on the left hind limb after the second round of FBZ injections. Lesions were treated (as described above) and resolved. We recovered a single female GW from the left hind leg, which appeared flat, lacking robustness and a normal, healthy GW appearance. Larvae were recovered, exhibiting a sluggish swimming movement. Larvae were exposed to copepods and infection status checked on October 10, 2019. We found a single copepod (1/145) infected with a single GW larva. This larva did not develop to L3 stage in the copepod and was dead at 14 days post infection (dpi).

Ferret 3 (E5035):

Ferret 3 received FBZ treatment, then was euthanized and necropsied on October 23, 2019 (350 dpe). Of note, this individual ferret developed sterile abscesses on the left hind limb after the second round of FBZ injections. Lesions were treated (as described above) and resolved. Three GWs were recovered from ferret 3: a gravid female worm was found on the right front limb, a single male was recovered from the right intercostal area, and another single male was found in the right inguinal area. Larvae were recovered from the female worm and exhibited more active swimming movement compared to the worms recovered from ferrets 1 and 2. After a few hours, while larvae were exposed to copepods, swimming behavior of larvae slowed. Copepods were checked for infection October 28, 2019, and no larvae were present in copepods (0/154). All copepods exposed to larvae from ferret 3 were checked again November 12 to verify the lack of infection, and no larvae were found.

Ferret 4 (E5030):

Ferret 4 received FBZ treatment, then was euthanized and necropsied on November 12, 2019 (371 days post exposure). Of note, this individual ferret developed sterile abscesses on the left hind limb after the second round of FBZ injections. Lesions were treated (as described above) and resolved. A single female worm was found coiled in the left rear leg. The worm was in a purulent cyst and was partially degraded. No larvae were recovered.

Ferret 5 (E5027):

Ferret 5 received FBZ treatment, then was euthanized and necropsied on November 20, 2019 (379 days post exposure). A subcutaneous worm was visible running transversely across the abdomen beginning at 9 months post exposure. This worm did not migrate through the course of gestation. Upon necropsy, this female worm was found to be in poor condition. Larvae were removed from the worm, but all were in a state of degradation. A second female worm was in the left inguinal region and appeared more robust and healthier than the first. Upon removal and larval examination, few (< 10) fully formed, dead L1s were observed, and the uterus of the worm was filled with a granular mass. No larvae were available to expose to copepods.
